# Opportunities to tackle structural racism and ethnicity-based discrimination in recovering and rebuilding from the COVID-19 pandemic

**DOI:** 10.1038/s41467-022-30791-w

**Published:** 2022-06-14

**Authors:** Natalia Linos, Mary T. Bassett, Alejandra Salemi, Margareta Matache, Konstantinos Tararas, Rodney Kort, Susana Gomez, Michela Zaghi, Rosemary Lane, Brianna Harrison, Karin Lucke, Gianna Sanchez, Anne Althaus, Mirna P. Amaya, Theadora Swift Koller

**Affiliations:** 1 FXB Center for Health and Human Rights, Harvard University, 677 Huntington Avenue, Boston, MA 02115 USA; 2 United Nations Educational, Scientific and Cultural Organization, 7 Place de Fontenoy, 75352 Paris Cedex 07, France; 3 World Health Organization, Avenue Appia 20, 1211 Geneva, Switzerland; 4 United Nations Department of Economic and Social Affairs, New York, NY 10017 USA; 5 United Nations Development Coordination Office, 405 East 42nd Street, New York, NY 10017 USA; 6 Office of the United Nations High Commissioner for Human Rights, New York, NY 10017 USA; 7 International Migration Law Unit, International Organization for Migration, 17 Route des Morillons, 1211 Geneva 19, Switzerland

**Keywords:** Public health, Decision making

## Abstract

The impact of COVID-19 has been disproportionately felt by populations experiencing structural racial- and ethnicity-based discrimination. Here, the authors describe opportunities for COVID-19 response and recovery efforts to help build more equal and resilient societies.

## Teaser

The impact of COVID-19 has been disproportionately felt by populations experiencing structural racial- and ethnicity-based discrimination. Here, we describe opportunities for COVID-19 response and recovery efforts to help build more equal and resilient societies, through investments in: (i) interventions focused on explicitly addressing racial and ethnicity-based discrimination; (ii) interventions supporting the delivery of universal services, and in ways that address compounding and intersecting drivers of exclusion and marginalization; and (iii) cross-cutting enabling measures, such as participatory mechanisms and data disaggregation.

More than two years since the first SARS-CoV-2 infections were reported, the COVID-19 pandemic remains an acute global emergency^1^. While many countries have successfully vaccinated significant portions of their populations, stark global inequities remain with imbalance in the global distribution of vaccines^2,3^, and potential new variants could further threaten the ability of governments to recover from the inter-connected health, economic, and broader human rights crises. Within countries, the impact of COVID-19 has been disproportionately felt by populations experiencing structural racial- and ethnicity-based discrimination. Indeed, where disaggregated epidemiologic data are available, COVID-19 morbidity and mortality rates are often significantly higher among people of African descent, indigenous peoples, and ethnic groups or other minoritized groups experiencing discrimination^4–6^. This reflects what social epidemiologists have long recognized: disease distribution is patterned by structures of disadvantage, inequality, marginalization and discrimination that often have historical roots and still have present-day manifestations^7^. Importantly, “race” is purely a social construct that has no biological basis, and documented racial inequities are due to racism not genetics or biology.

Ethnicity-based and racial discrimination intersects with other forms of exclusion based on income, occupation, gender identity and expression, sexual orientation, disability, religion or belief, language, migratory status, rural/urban status and more, and is unlikely to exist in only one sector. Beyond health inequities, COVID-19 racial and ethnicity-based inequities have been documented across employment, education, housing and food insecurity, among other domains^8^. There are different pathways – from differential exposure, to inequitable access to health services, to uneven socioeconomic impacts of control measures, that can be further studied to inform policymaking, planning and programming^9^. Yet, these unequal impacts and human rights violations are not inevitable; neither is a spike in xenophobia and hate speech^10^.

As countries work to respond to the ongoing COVID-19 pandemic and address both health challenges and broader socio-economic impacts, attention must be given to tackling racial and ethnic discrimination. With this in mind, and consistent with the UN Secretary General’s Call to Action for Human Rights^11^, members of the United Nations Sustainable Development Group released a report on opportunities to address racial- and ethnicity-based discrimination^12^.

The report was developed through a consultative process that began in late 2020 through early 2021, involving UN senior executives and technical staff, civil society, public health practitioners and human rights experts. It identified three strategic approaches to addressing racial and ethnicity-based discrimination in COVID-19 response and recovery efforts. These are: (i) interventions explicitly tackling racial and ethnicity-based discrimination, including improving anti-discrimination and redress mechanisms; (ii) interventions supporting the delivery of universal services, but in ways that address compounding and intersecting drivers of social exclusion and draw on the framework of proportionate universalism^13^; and (iii) investments in cross-cutting enabling measures, such as participatory mechanisms and data disaggregation (see Table 1 which is replicated from the report).

**Table 1:** Summary of potential entry points to address racial- and ethnicity-based discrimination, replicated from UN report.

Action area 1: Interventions explicitly tackling racial and ethnicity-based discrimination
1.1: Tackle xenophobia, racist disinformation, hate speech and media stereotyping.
1.2: Address law enforcement culture and practices.
1.3: Adopt special measures, including affirmative action and targeted financial assistance.
1.4: Support the rights of indigenous peoples.
1.5: Strengthen anti-discrimination measures and grievance redress mechanisms.
1.6: Strengthen autonomous national institutions or create new mechanisms ensuring access to justice and redress.
1.7: Track and address triggers linked to inter-ethnic violence and atrocity crimes, including genocide.
1.8: Invest in strong social inclusion policy.
Action area 2: Interventions addressing compounding and intersecting drivers of social exclusion
2.1: Ensure inclusive and equitable public policy and programming across domains, through the adoption of a human rights-based approach (HRBA).
2.2: Improve infrastructure, services and local inclusive governance (including community engagement mechanisms) in areas with high levels of multidimensional deprivation.
Action area 3: Critical transversal enablers and principles for a human rights-based approach (HRBA)
3.1: Ensure meaningful political and civic participation of communities experiencing discrimination.
3.2: Enable data disaggregation and inequality monitoring efforts, with appropriate protection safeguards.
3.3: Ensure universal right to birth registration and legal identity and invest in vital statistics and civil registration.
3.4: Dedicate sufficient resources through equity-oriented and participatory budgeting, and support to civil society.
3.5: Invest in accurate communications, elevating messages of solidarity, tolerance and inclusion.

Outcome report “Addressing structural racial and ethnicity-based discrimination: Key action areas for COVID-19 recovery plans” by the United Nations Sustainable Development Group (UNSDG) Task Team on Leave No One Behind, the Human Rights and the Normative Agenda, co-led by the Office of the United Nations High Commissioner for Human Rights (OHCHR), the United Nations Educational, Scientific and Cultural Organization (UNESCO) and the World Health Organization (WHO), under the auspices of the UNSDG. Table 1 depicts opportunities for COVID-19 response and recovery efforts to help build more equal and resilient societies, through investments in three synergistic action areas. Action area 1 describes interventions explicitly tackling racial and ethnicity-based discrimination. Action area 2 covers interventions addressing compounding and intersecting drivers of social exclusion. Action area 3 addresses cross-cutting enabling measures.

While not exhaustive and recognizing that entry-points would need to be tailored to national circumstances and developed in consultation with impacted communities, these mutually reinforcing entry-points together offer a framework to address discrimination directly through targeted measures, and indirectly through universal measures that will have amplified impacts on disadvantaged populations. This framework is currently being used in a capacity-building programme for United Nations Country Teams to support national policies and programmes that result in more just, equal and resilient societies. To date, thirteen UN Country Teams have participated in the programme and a training-of-trainers is planned for late 2022 to reach more, under the umbrella of the UN Network on addressing Racial Discrimination and the Protection of Minorities.

## Explicitly tackle racial and ethnicity-based discrimination

COVID-19 made clear that there is a need for explicit policies to tackle racial and ethnicity-based discrimination in the immediate term, to address current pressing needs, and in the long term. The longstanding impact of colonialism, theft of land, resources and exclusionary actions, for example, left indigenous peoples more vulnerable to COVID-19. Indigenous peoples already faced numerous obstacles to equitably accessing health services, including because of geographical barriers posed by the organization of provider networks, difficulty affording medical care, or difficulties in accessing culturally appropriate care that is in their own language and that respects their cultural beliefs, traditions and practices^14^. During the COVID-19 pandemic the world also witnessed a rise in episodes of xenophobia, hate speech, abuses, assaults, harassment, scapegoating, stigmatization and increase in excessive immigration control measures^15^.

Countries can use this opportunity of heightened sensitivity to health inequities to adopt or strengthen existing anti-discrimination laws and policies that align with international human rights standards. These should extend beyond health care to address discrimination in the workplace, housing, education, law enforcement and border control sectors, among others. States must meet their obligations to respect, protect and fulfill the rights of everyone guaranteed under the *International Convention on the Elimination of all Forms of Racial Discrimination* by, for example, equipping existing autonomous national institutions to guarantee protection, effective implementation of anti-discrimination laws and policies, and access to justice for individual or collective complaints.

Accurate communications and public messages centered on solidarity, tolerance, and inclusion are needed to combat scapegoating, ‘othering’ or victim-blaming narratives that continue to exacerbate harm. Just as with past epidemics which have often resulted in human rights abuses and discriminatory enforcement of public health measures, as well as segregationist public health strategies^16^, concerns were raised around emergency measures inappropriately targeting groups, including migrants, refugees and asylum seekers, and the excessive and selective use of police and military for enforcement^17^. For example, longstanding anti-Roma racism in Europe, including violent and targeted oppression over centuries, resurfaced through inflammatory rhetoric and scapegoating during COVID-19^18^.

## Addressing compounding and intersecting drivers of exclusion

COVID-19 made inequities experienced by persons facing ethnicity-based and racial discrimination more visible, but unfair and discriminatory practices in education, housing, labor markets, the criminal justice system, to name a few, are often overlapping and compounding, and did not emerge simply as a result of the pandemic. Universal and rights-based strategies such as temporary moratoria on evictions^19^, expanded social protection nets, enhanced labor protections including for workers in the informal economy, expansion of free Wi-Fi and open education resources, release and decarceration efforts – among other measures – can have a differential positive impact on populations experiencing racial and ethnicity-based discrimination. This is because structural racism acts to skew distributions such that excluded and marginalized groups are over-represented among those working in the informal economy, those facing housing insecurity, those deprived of education, those living in poverty and those facing obstacles to justice. An intersectional analysis to comprehensively address the multiple and overlapping structural drivers of inequality, for example with regards to gender identity, religion, ableness, or citizenship status, in addition to race and ethnicity, is essential in COVID-19 recovery plans.

Place-based strategies^20^, including neighborhood and community-based interventions, can similarly help mitigate the disproportionate impact of COVID-19 in geographic regions that have historically been underserved and contain large numbers of people that may have experienced racial and ethnicity-based discrimination. For example, investments in infrastructure, including health infrastructure, can help to ensure basic minimum needs for shelter, health, water, sanitation and hygiene are being met during and following the pandemic, while also generating employment opportunities in the immediate term for residents of underserved areas.

## Invest in enabling and human rights-based approaches

Although there are both direct and indirect strategies through which plans to build forward better from the COVID-19 pandemic can begin to address structural racism and ethnicity-based discrimination, as described above, these actions will only be successful if they address power inequities and are developed through participatory and inclusive approaches, ensuring historically excluded communities have decision-making power and adequate resources. COVID-19 response and recovery plans could earmark resources for civil society organizations working to address discrimination, social exclusion and human rights.

In the immediate term, investments in systems to disaggregate data by race and/or ethnicity, as well as other dimensions that can help address the intersectional impact on particular groups, are needed. It is essential that these are accompanied by investments in data protection. These investments and other equity analysis tools can help unpack inequalities across groups both as a direct result of COVID-19 and because of unevenly distributed impacts of pandemic-related measures and uneven socioeconomic consequences. Importantly, mechanisms for transparent data reporting that allow for public accountability will be critical. In many contexts, investment in vital statistics and civil registration will also be needed, since the pandemic highlighted that many people in need of emergency social protection were unable to access benefits because they lacked proper identification^21^. Similarly, efforts should be made to ensure that the needs of those with precarious legal identity status are also met.

## Conclusion

Inequalities in who gets sick and who suffers long-term socioeconomic consequences during and following a pandemic are largely shaped by unequal political power dynamics and how societies structure their economies, laws and policies and broader governance mechanisms. This tragic pandemic has further shone the light on grave and historic injustices and inequalities, both within and across countries, and should be an impetus for countries to strengthen their efforts to address systemic racism and ethnicity-based discrimination. Lessons learned from the COVID-19 pandemic also include the importance of prevention and the high cost of inaction. Parallels can easily be drawn around the need for urgent and concerted action to address the climate emergency if we are to prevent the unnecessary and unequal health impact of climate change^22^. This opportunity should not be wasted.

## References

[1] World Health Organization. *Strategic preparedness, readiness and response plan to end the global COVID-19 emergency in 2022* (World Health Organization, Geneva, 2022) (WHO/WHE/SPP/2022.01).

[2] Kavanagh MM, Gostin LO, Sunder M (2021). Sharing technology and vaccine doses to address global vaccine inequity and end the COVID-19 pandemic. JAMA.

[3] World Health Organization. *Vaccine equity campaign*. (WHO, 2021). https://www.who.int/campaigns/vaccine-equity.

[4] Shadmi, E. et al. Health equity and COVID-19: global perspectives. *Int. J. Equity Health* **19**, 104 (2020). 10.1186/s12939-020-01218-z PMC7316580 32586388

[5] Sze, S. et al. Ethnicity and clinical outcomes in COVID-19: A systematic review and meta-analysis. *EClinicalMedicine* **29**, 100630 (2020). 10.1016/j.eclinm.2020.100630 PMC7658622 33200120

[6] Yaya, S., Yeboah, H., Charles, C. H., Otu, A. & Labonte, R. Ethnic and racial disparities in COVID-19-related deaths: counting the trees, hiding the forest. *BMJ Global Health* 5, e002913 (2020). 10.1136/bmjgh-2020-002913 PMC7298686 32513864

[7] Bailey ZD (2017). Structural racism and health inequities in the USA: evidence and interventions. Lancet.

[8] Paremoer L, Nandi S, Serag H & Baum F. Covid-19 pandemic and the social determinants of health. *BMJ* **372**, n129 (2021). 10.1136/bmj.n129 PMC7842257 33509801

[9] Katikireddi SV (2021). Unequal impact of COVID-19 crisis on minority ethnic groups: a framework for understanding and addressing inequalities. J. Epidemiol. Community Health.

[10] Krieger N (2003). Does racism harm health? Did child abuse exist before 1962? On explicit questions, critical science, and current controversies: an ecosocial perspective. Am. J. Public Health.

[11] United Nations. *The highest aspiration: a call to action for human rights* (United Nations, 2020).

[12] World Health Organization. *Frontier dialogue consultations on addressing structural racial and ethnicity-based discrimination: key action areas for COVID-19 recovery plans* (World Health Organization, 2021).

[13] Francis-Oliviero, F., Cambon, L., Wittwer, J., Marmot, M. & Alla, F. Theoretical and practical challenges of proportionate universalism: a review. *Pan Am. J. Public Health*. **44**, e110 (2020). 10.26633/RPSP.2020.110 PMC7556407 33088291

[14] International Labour Organization. *COVID-19 and the world of work: a focus on indigenous and tribal peoples* (International Labour Organization, 2020).

[15] Guadagno, L. *Migrants and the COVID-19 pandemic: an initial analysis*. Migration Research Series N° 60, 5 (International Organization for Migration (IOM), 2020).

[16] Finn BM, Kobayashi LC (2020). Structural inequality in the time of COVID-19: urbanization, segregation, and pandemic control in sub-Saharan Africa. Dialogues Hum. Geogr..

[17] Linos, N. & Bassett, M. T. *Public health calls for solidarity, not warfare* (Foreign Affairs, 2020).

[18] Matache, M., Leaning, J. & Bhabha, J. *The shameful resurgence of violent scapegoating in a time of crisis* (Open Democracy, 2020).

[19] Nande, A. et al. The effect of eviction moratoria on the transmission of SARS-CoV-2. *Nat. Commun.* **12**, 2274 (2021). 10.1038/s41467-021-22521-5 PMC8050248 33859196

[20] Henson, R. M. et al. Evaluating the health effects of place-based slum upgrading physical environment interventions: a systematic review (2012–2018). *Social Sci. Med.* **261**, 113102 (2020). 10.1016/j.socscimed.2020.113102 PMC7611465 32739786

[21] World Justice Project. *The COVID-19 pandemic and the global justice gap* (World Justice Project, 2020).

[22] World Health Organization. *Climate change and health* (World Health Organization, 2020).

